# Mycotoxin Challenge in Dairy Cows: Assessment of the Efficacy of an Anti-Mycotoxin Agent by Adopting an In Vitro Rumen Simulation Method

**DOI:** 10.3390/toxins16110490

**Published:** 2024-11-13

**Authors:** Erica Fiorbelli, Marco Lapris, Michela Errico, Antonella Della Badia, Insaf Riahi, Gabriele Rocchetti, Antonio Gallo

**Affiliations:** 1 Department of Animal Science, Food and Nutrition (DIANA), Faculty of Agricultural, Food and Environmental Sciences, Università Cattolica del Sacro Cuore, 29122 Piacenza, Italy; erica.fiorbelli@unicatt.it (E.F.); marco.lapris@unicatt.it (M.L.); michela.errico@unicatt.it (M.E.); gabriele.rocchetti@unicatt.it (G.R.); 2 Technical Department, BIŌNTE Nutrition S.L., 43204 Reus, Spain; antonella.dellabadia@bionte.com (A.D.B.); insaf.riahi@bionte.com (I.R.)

**Keywords:** rumen, mycotoxin detoxifier, adsorbent material, phytogenics, post-biotic compounds

## Abstract

To protect ruminants from the harmful effects of mycotoxins, anti-mycotoxin agents can be added to the dietary ration, thus guaranteeing animal health and production. Therefore, the objective of this study was to evaluate the in vitro ruminal initial sequestration (weak binding) and subsequent desorption (strong binding) of an anti-mycotoxin agent based on a mixture of adsorbing material, turmeric and milk thistle extracts and yeast-based components to adsorb or bio-convert aflatoxins (AF), fumonisins B1 and B2 (FB), trichothecene deoxynivalenol (DON), T-2 and HT-2 toxins, and zearalenone (ZEN). Two doses were tested: Dose 1 simulated 30 mg/cow/d, while Dose 2 simulated 90 mg/cow/d of the anti-mycotoxin agent. Each treatment involved three analytical replicates at each of three incubation times (1, 4, and 24 h post-incubation), with two independent experimental runs providing experimental replicates. Analytical methods, including UHPLC-HRMS and multivariate analyses, were used to both quantify mycotoxin concentrations and reveal dose-dependent reductions, with statistical validations indicating significant changes in mycotoxin levels across both dose and time. The results indicated that the anti-mycotoxin agent was able to highly bind AFB1, T2, and HT-2 toxins since its concentration was always under the limit of detection (<1 ppb). Regarding ZEN (weak binding mean: 94.6%; strong binding mean: 62.4%) and FBs (weak binding mean: 58.7%; strong binding mean: 32.3%), orthogonal contrasts indicated that the anti-mycotoxin agent was able to effectively bind these toxins using Dose 1 (*p* < 0.05). This finding suggests that Dose 1 may be sufficient to achieve the targeted effect and that a further increase does not significantly improve the outcome. Regarding DON, a strong linear relationship was observed between dose and adsorption. However, the complex interactions between the mycotoxin, the ruminal environment, and the anti-mycotoxin agent made it difficult to establish a clear dose–effect relationship (*p* > 0.10). UHPLC-HRMS analysis identified over 1500 mass features in rumen samples, which were further analyzed to assess the effects of the anti-mycotoxin agent. Hierarchical clustering analysis (HCA) revealed significant changes in the untargeted metabolomic profiles of samples treated with mycotoxins compared to control samples, particularly after 24 h with the anti-mycotoxin treatments. Clear differences were noted between strong binding and weak binding samples. Further analysis using orthogonal partial least squares discriminant analysis (OPLS-DA) highlighted distinct metabolomic profiles, with stronger predictive ability in the strong binding group (Q^2^ cumulative value of 0.57) compared to the weak binding group (0.30). The analysis identified 44 discriminant compounds in the strong binding model and 16 in the weak binding model. Seven compounds were common to both groups, while silibinin, known for its antioxidant and anti-inflammatory properties, was found among the unique compounds in the weak binding group. Overall, the findings suggest that both doses of the anti-mycotoxin agent significantly influenced the chemical profiles in the rumen, particularly enhancing the binding of mycotoxins, thereby supporting the role of phytogenic extracts in mitigating mycotoxin effects.

## 1. Introduction

Mycotoxins are identified as a group of toxic compounds produced by several filamentous fungi in the genera of *Aspergillus*, *Fusarium*, *Penicillium,* or *Alternaria* [1,2,3]. Their high stability can be attributed mainly to their chemical structure, but their prevalence is enhanced by the lack of application of good agricultural practices (GAPs) and, in recent years, by climate change [4]. Altogether, these factors lead to these compounds being incredibly widespread in raw materials and finished feeds for animal nutrition. In fact, it has been estimated that at a global level, between 30 and 100% of food and feed samples are co-contaminated with mycotoxins [4,5,6].

Mycotoxicosis results in large economic losses for farmers because it not only negatively impacts animal health (e.g., decrease in productivity) but also increases farm variable costs (e.g., the costs for destroying contaminated feed) [7,8]. There are several factors that play a crucial role in mycotoxin exposure in animal production, such as the selection of raw materials included in the animal diet to meet the nutritional requirements of each species [9]. When it comes to fulfilling animal energy requirements, the choice falls on cereal grains, which account for the majority of animal diets and feedstuffs [10,11,12]. It is well-known that cereal grains represent the main source of mycotoxins exposure, primarily due to the high incidence of deoxynivalenol (DON), zearalenone (ZEN), aflatoxins (AFs), T-2 toxin, and ochratoxin A (OTA) [13,14,15]. Corn, soy, and wheat represent the main raw materials included in monogastric species diets; thus, the general idea is that only these species can suffer from mycotoxin effects. However, as outlined by Gallo et al. [12], ruminant diets comprise a wide range of raw materials, such as forage, silage, or by-products, which can increase the mycotoxins risk level in these species [16]. Another condition that could contribute to mycotoxin exposure is linked to environmental factors; in fact, temperature and humidity play a pivotal role in mold growth, especially during the storage phases of plants intended for animal feeds [17,18].

As extensively reviewed by Ogunade et al. [19], mycotoxin contamination can originate in both pre-harvest and post-harvest phases, thus contributing to a final ration contaminated by several less-studied mycotoxins [20]. To overcome mycotoxin exposure in animal production, a lot of strategies have been developed. The application of GAP has always been one of the most effective solutions. Crop rotation, tillage, or the use of less susceptible cultivars represent some of the pre-harvest GAPs; while post-harvest GAPs include a reduction in the humidity level before and during storage or ensuring that the silos are well packed to preserve anaerobic conditions [7,8,21]. However, it must be considered that the production of mycotoxins is impossible to avoid, as there are factors that are beyond environmental control (e.g., the soil type) [22,23]. For this reason, different treatments have been employed, either directly on the feed (e.g., physical treatments on the grain) or solutions that act directly on the animal (e.g., mycotoxin agents) to manage mycotoxin contamination [8,24,25].

Mycotoxin agents are used less in ruminants since this species is able to metabolize mycotoxins in their other or less toxic forms through ruminal detoxification processes [26,27]. Debevere et al. [28] simulated the disappearance of DON and other less-studied mycotoxins (e.g., nivalenol and enniatin B) in the ruminal fluid of lactating cows and showed that this toxin is almost completely converted into the less toxic metabolite diepoxy-DON (DOM-1) in ruminal normal conditions. However, this mechanism could be altered by several factors: low pH (SARA conditions) [29]; functional ruminal microbiota (non-ruminants vs. ruminants) [30]; production level (e.g., high-producing cows) [28]; the total level of mycotoxins contamination; and the synergistic effects between them [12]. These factors can detrimentally increase mycotoxin effects on ruminants since some of them can pass through the ruminal tract unchanged and, thus, exert systemic negative effects [31]. Also, mycotoxins are cytotoxic, hepatotoxic, nephrotoxic, and immunosuppressive [32,33,34]. While the mechanisms behind these responses are not yet fully understood, the common indicators frequently involve ruminal or gut dysbiosis, the increased permeability of the rumen or gut linings, and gut damage [35]. The direct negative effects described refer to alterations linked to the gastrointestinal and reproductive tract, as well as to the mammary gland [36,37]. Altogether, these effects are responsible for a decrease in feed intake, milk quality, production, and fertility [12,38,39,40], as well as increases in mastitis incidence and milk contamination [12,39,40].

In 1960, with the discovery of aflatoxin b1 (AFB1), the challenge was to find solutions to mitigate the effects of this mycotoxin [41]. Numerous studies have shown that certain clays, such as bentonite and sepiolite, are able to bind not only AFB1 but also other AFs (AFB2, AFG1, AFG2; AFM1) due to their structure and affinity [2,24,42]. Similarly, the yeast cell wall (YCW) showed a binding capacity towards ZEN [43,44]. Over time, however, although the toxic effects of aflatoxins have been neutralized, it has been found that other mycotoxins could exert harmful effects on animal health [45]. For example, liver damage and oxidative stress, commonly associated with toxins such as DON, T-2 toxin, and FBs, are fundamental processes underlying numerous diseases [46]. Therefore, several natural solutions have been tested to protect animals from the hepatic and intestinal alterations produced by mycotoxins.

In order to counteract the negative effects of these contaminants on animal health, the European Union approved the use of a group of additives defined as “substances that can suppress or reduce the absorption, promote the excretion or modify their mode of action” [47]. At the same time, the European Food Safety Authority issued a review discussing mycotoxin-detoxifying agents utilized as feed additives [48], which encompassed their mechanisms of action, effectiveness, and safety for feed and food consumption. These additives are classified according to their mode of action, which includes adsorption, referring to their ability to bind mycotoxins to their surface, and biotransformation, which involves the degradation of mycotoxins into non-toxic metabolites [48]. In recent years, a novel approach has emerged for managing mycotoxins in animal diets by incorporating specific additives. This strategy utilizes substances such as phytogenics to enhance the protective effects of anti-mycotoxin agents in animal nutrition [49,50]. Phytogenics represents a natural alternative with a strong bioprotective capacity against mycotoxin exposure and has been shown to improve growth performance, nutrient digestibility, antioxidant capacity, and intestinal barrier integrity [51,52].

Among the most effectively studied phytogenic extracts, curcumin and silymarin extracts are those which show a very potent effect on animal health status. Curcumin [1,7-bis(4-hydroxy-3-methoxyphenyl)-1,6-heptadiene-3,5-dione] has received considerable interest in animal production due to its different pharmacological activities, which include antioxidant [53], antimicrobial [54], and anti-inflammatory properties [55,56,57]. Conversely, silymarin extract bioactive constituents exhibit antibacterial activity through their hydroxyl group, which can bind to bacterial membrane proteins, leading to the leakage of vital cellular components [58,59]. Both extracts have been shown to be highly effective in inhibiting MAPK pathways, including Janus kinase/signal transducers and activators of transcription (JAK/STAT), nuclear factor κB (NFκB), p38 and organic anion-transporting peptides (OATPs). This anti-mycotoxin agent presents all these ingredients in its formulation, and its efficacy was previously tested in vivo in sows and piglets [60,61]. However, no study has been conducted on ruminants. For all these reasons, the aim of this study was to test the efficacy of an anti-mycotoxin agent, considering an in vitro ruminal simulating method against AFB1, T-2, HT-2, DON, ZEN, and FBs. The tested anti-mycotoxin agent contains two sources of phytogenics (turmeric and milk thistle extracts), which are known to reduce oxidative stress and inflammation [62,63,64]. Curcumin is among the most studied phytogenics as it has demonstrated a wide range of pharmacological activities [65]. Regarding mycotoxin mitigation, curcumin has been demonstrated to reduce oxidative liver damage by selectively acting on CYP450 isoenzymes and, thus, inhibiting the mycotoxin hepatic activation of toxic metabolic forms [62,66]. The second phytogenic included in the anti-mycotoxin agent formula is the milk thistle extract, which is known to have a strong antioxidant activity due to nuclear DNA/RNA-mediated effects through suppression of translocation and NF-κB binding, thus minimizing the cellular damage and oxidative stress induced by mycotoxins [67].

Finally, some post-biotic effects can be provided by yeast products characterizing the tested anti-mycotoxin agent. In this regard, among the compounds usually used in anti-mycotoxin agents, the yeast cell wall has demonstrated the ability to adsorb mycotoxins [68]. The composition of the yeast cell wall is characterized by lipids, chitins, proteins, and polysaccharides (e.g., mannan and glucans). The structure of β-D-glucans and the distribution between β-(1,6)-D-glucan and β-(1,3)-D-glucan has been shown to cope with AFB1, T-2, OTA, ZEN, and DON exposure due to their complex structure [64,68,69]. The binding process is enhanced by non-covalent hydrogen bonds and hydrophobic or ionic interactions capable of adsorbing the mycotoxins [70,71]. However, the effectiveness of the yeast cell wall is optimized at a near-neutral pH, as opposed to alkaline conditions. Acidic environments, such as those in the digestive system, promote the functionality of glucans, which is one reason why yeast and its derivatives are considered for inclusion in feed additives [72].

## 2. Results and Discussion

In the scientific literature, there are limited studies on the efficacy of anti-mycotoxin agents in ruminants due to the high individual variability within this species and their apparent greater resistance to mycotoxins [1,12]. However, as certain factors have been shown to impair ruminants’ resistance to mycotoxins, it is crucial to include specific solutions in their diet to mitigate these adverse effects [28].

The evaluated anti-mycotoxin agent claims to be effective against mycotoxins through its triple-action mechanism. This encompasses the adsorption process and the mitigation of mycotoxin effects via hepatoprotective, antioxidant, and anti-inflammatory actions, which result from incorporating phytogenic extracts into the formulation. In this work, we evaluated the efficacy of an anti-mycotoxin agent in reducing the ruminal concentration of mycotoxins in dairy cows.

### 2.1. In Vitro Gas Production

**Figure 1:**
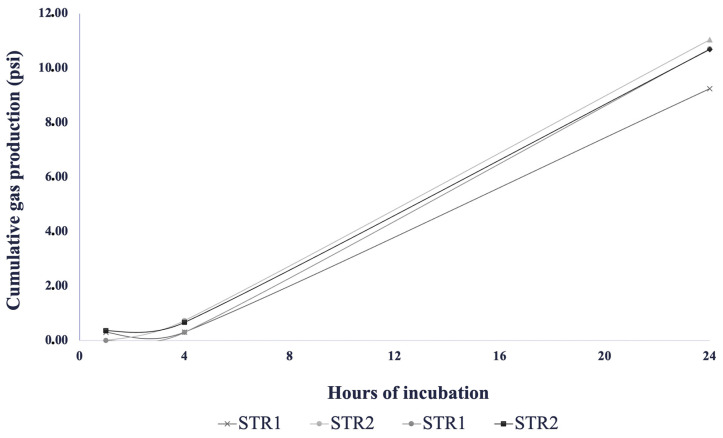
Gas production of the standard sample (STR) in run 1 and run 2.

**Figure 2:**
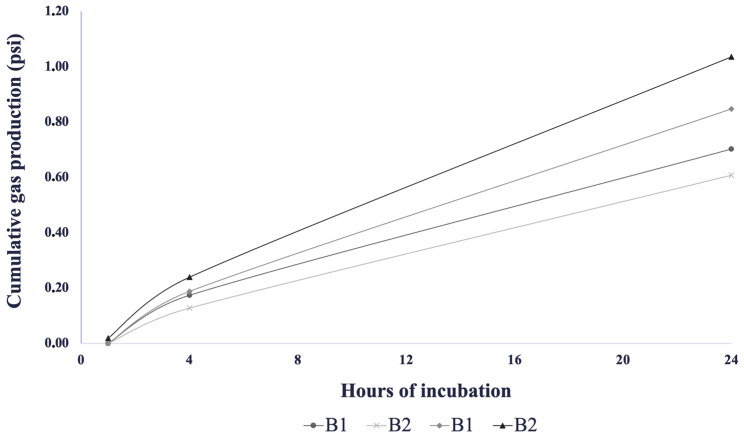
Gas production of the blank sample in run 1 and run 2.

Figure 1 and Figure 2 show the gas production of the replicate of standards and blank samples in pound-per-square-inch (PSI) units in all the experimental runs.

During run 1, the means of the cumulative gas of standard samples were 0.150 ± 0.21, 0.522 ± 0.29, and 10.14 ± 1.26 psi, respectively, for 1, 4, and 24 h of incubation. While for run 2, the means were 0.180 ± 0.25, 0.482 ± 0.02, and 10.69 ± 0.01 psi, the final standard deviation of the two runs for each replicate was less than one, supporting the conclusion that the experimental fermentations were conducted as expected. Regarding the blank samples, the means of cumulative gasses were 0 ± 0, 0.152 ± 0.03, and 0.656 ± 0.07 psi, respectively, for 1, 4, and 24 h of incubation for run 1; for run 2, the means were 0.01 ± 0.01, 0.215 ± 0.04 and 0.942 ± 0.13 psi.

As shown in Figure 1, a long lag phase (latent phase) was observed in the in vitro gas production test of standard samples; this could be attributed to several factors related to the nature of the substrate (i.e., corn–barley flakes) used in the assay. Generally, a smaller particle size offers a larger surface area, which should theoretically result in a shorter lag phase as microbes can colonize the substrate more easily. However, we used a purified corn sample with a variable structure (i.e., including larger or more compact particles), thus potentially leading to uneven fermentation and a delayed start of fermentation activity. Also, the rumen microbial community might need time to adapt to the substrate. Although corn and barley are common in animal feeds, under our in vitro conditions, variability in the physical and chemical structure of the flaked material could result in a lag in microbial enzyme production or attachment. This adaptation phase is commonly reflected in a longer lag phase. Corn–barley substrates with a high starch content can also lead to rapid acidification once fermentation starts, which might initially suppress fermentation, elongating the lag phase. Besides this starting behavior, we can also assert that the buffered rumen fluid without a substrate had a late capacity to ferment during the first hour. The final standard deviation of the two runs for each replicate was <1, thus indicating that the experimental fermentations were conducted as anticipated.

### 2.2. Anti-Mycotoxin Agent Efficacy

Mycotoxin contamination represents a significant challenge in agricultural environments as its complete prevention is hard to avoid [73]. However, numerous studies have been conducted to identify compounds that are able to mitigate the toxic effects of mycotoxigenic fungi on animal health [74]. The EFSA reported that substances used to reduce mycotoxin contamination in feed can be divided into two categories: additives that suppress or reduce the absorption and/or enhance the excretion of mycotoxins and additives that alter the chemical structure of mycotoxins to modify their mode of action [75]. The former concept refers to clay-based adsorbents, which have been demonstrated to be highly efficient in adsorbing several mycotoxins due to their chemical bonds between active sites in the toxin and clays [76]. The anti-mycotoxin agent tested contains bentonite, which, according to several studies, is able to efficiently adsorb AFs [12,77,78] and ZEN, OTA, and FBs [79,80]. This efficiency is attributed to the large surface area and high cation exchange capacity of the smectite group, enabling it to adsorb organic substances through the infiltration of both cations and polar molecules [48].

Among the nutritional strategies, the application of plant extracts and organic compounds has attracted considerable interest because of their antioxidant, hepatoprotective, immunostimulant, and antimicrobial effects [81]. In particular, turmeric and milk thistle extracts (both contained in the anti-mycotoxin agent) have shown numerous beneficial effects both in vitro and in vivo [82,83]. Starting with their ability to reduce the detrimental effects induced by mycotoxins, several authors showed that the turmeric extract was able to reduce the oxidative stress and inflammation induced by mycotoxins through the inhibition of MAPK pathways, specifically the Janus kinase/signal transducers and activators of transcription (JAK/STAT), nuclear factor κB (NFκB), and p38 pathways [84,85]. On the other hand, silymarin has been shown to act as an inhibitor of the OATP, preventing the entry of DON into hepatic and intestinal cells, thus protecting the cells from the harmful effects of this mycotoxin [86].

Phytogenics are widely recognized for their antimicrobial properties, which can modulate rumen microbiota composition [87]. Several studies have demonstrated the in vitro capacity of curcumin and silymarin to reduce methane production [82,88], while other research has highlighted its antioxidant and anti-inflammatory effects, which can lead to an increased milk yield [83,87]. Regarding organic components, yeast cell walls have been widely used as an alternative to antibiotics, as they can enhance the intestinal barrier, absorb pathogens, and promote cytokine release. In ruminants, various studies have shown that YCW supplementation enhances milk yield [89] and supports ruminal development [90].

Table 1 provides detailed information on the results obtained with the tested anti-mycotoxin agent under in vitro rumen-simulating conditions. By using rumen fluid and conditions that mimic the natural rumen environment, our in vitro gas production test provides a more realistic assessment of how anti-mycotoxin agents interact within the animal’s digestive system. In this regard, mycotoxins and anti-mycotoxin agents might behave differently when exposed to live microbial populations, enzymes, and fermentation gasses compared to purely chemical environments (based on different pH values). Therefore, this approach allowed us to calculate the initial sequestration (weak binding) and desorption (strong binding) of the anti-mycotoxin agent for the different mycotoxins under investigation. As a general rule, weak binding should be higher than 80%, whereas strong binding should be lower than 20% [91,92]. The table outlines the treatment, mycotoxin, control (CTR), Dose 1, Dose 2, and the hour of incubation (1 h, 4 h, 24 h) for each mycotoxin (DON, FBs, ZEN). Under our experimental conditions, we found fluctuating values as a result of the mycotoxins considered (i.e., AFB1, ZEN, FBs, DON, T2, and HT-2). Under our experimental conditions, the AFB1, T2, and HT-2 concentrations always resulted in values under the LOD (limit of detection <1 ppb) for all samples, thus suggesting the high capacity of these products to bind to mycotoxins, likely due to the strong ability of the adsorbent material to block these compounds.

Looking at Table 1, we can see significant differences (*p* < 0.05) between the estimated marginal means mainly for weak bindings, while only FBs registered a significant trend when considering the strong binding effect. Regarding the effect on ZEN, the utilization of orthogonal contrasts (Table S1) revealed that while increasing the dose initially improved ZEN weak binding, the benefit may start to diminish at higher levels, considering the significance of both linear and quadratic trends (*p* < 0.01). This result implies that Dose 1 may be sufficient to achieve the desired effect, and increasing the dose further to Dose 2 does not significantly improve the outcome. This was confirmed by the statistical significance exclusively recorded for the interaction between Dose 1 *x* incubation time (*p* < 0.05). A similar trend was recorded for the weak binding of FBs (Table 1), showing a significant difference between Dose 1 and Dose 2 with the CTR; again, orthogonal contrasts showed that Dose 1 was sufficient to achieve the sequestering effect over time, although no significant correlations between Dose 1 × incubation time was measured (*p* < 0.05). The statistical analysis also outlined the major efficacy of Dose 1 when compared with Dose 2 to provide a strong binding effect, independently from the incubation time (*p* > 0.05). Additionally, a more significant quadratic trend (*p* < 0.01) was outlined for the strong binding of FBS, indicating that the effect changes as the dose increases. Finally, the statistical model demonstrated a strong linear relationship (significant linear contrast) between dose and absorption (Table S1) for DON, but the quadratic term also played a role in this, thus suggesting no purely linear effect of the dose. The fluctuating strong binding values measured are likely due to the complex interplay of factors such as the mycotoxin structure, pH variation (due to microbial fermentation), binding interaction types (e.g., electrostatic forces, van der Waals forces, hydrogen bonding, and hydrophobic interactions), competition among toxins for binding sites, matrix effects (as promoted by some macromolecules of the corn–barley-flaked diet), and kinetics that were bound over the fermentation time. For example, AFs tend to have a high affinity for adsorbents due to their small size and hydrophobicity, while zearalenone and fumonisins may exhibit weaker or variable binding due to their larger molecular size or different types of polarity. Also, the dynamic pH of the rumen may lead to a desorption–rebinding process, influencing the percentage of strong binding. Each mycotoxin characterizing the contaminated diet may behave differently in response to these variables, resulting in a range of binding percentages from 20% to 80%. To better understand these trends, further investigation into the specific interactions between the anti-mycotoxin agent and each mycotoxin in the rumen-simulating environment is mandatory.

As extensively studied, AFB1 can pose a serious problem for animal health, besides its negative impact on farm profitability [93]. After ingestion, AFB1 is primarily converted into a hydroxylated metabolite known as aflatoxin M1 (AFM1), which is eliminated in urine, bile, and milk [94,95]. The carry-over of AFM1 into milk represents one of the biggest problems for food safety and farm profitability [96]. The main characteristic of AFM1 is its high stability in all milk processing procedures (e.g., sterilization). The International Agency for Research on Cancer [41] classified AFB1, AFB2, AFG1, and AFG2 in Group 1 as carcinogenic agents, while AFM1 is included in Group 2B. To minimize the risk to public health related to the presence of this toxin in animal feed and food, the European Union established a benchmark dose for both feedstuffs and products of animal origin (Directive 2002/32/EC). Therefore, in order to comply with the maximum levels set in Europe for animal feed (for example, the EU sets 0.05 μg of AFM1 per kg in ruminant milk as the maximum residual level), specific substances are used in animal feed to minimize mycotoxin contamination [61]. However, clay alone cannot mitigate the effects induced by the AFB1. In fact, feed additives have been studied for their ability to prevent or mitigate aflatoxicosis in animal nutrition [97,98].

Another mycotoxin that could induce serious problems in dairy cows is zearalenone (ZEN). In fact, within the described effects, decreased fertility, abnormal estrus cycles, swollen vulvas, vaginitis, reduced milk production, and mammary gland enlargement are the most common findings reported in cattle [1,2], although alterations in ruminal fermentation have also been described [98,99]. Moreover, during ZEN contamination, suppression of the immune system has been described, mainly through the apoptosis of immune cells and organs, the up or down-regulation of important genes, and the alteration of oxidative pathways [100]. In contrast to the general idea of ruminal detoxification, when this mycotoxin is metabolized in the rumen, some metabolites can be produced that are even more estrogenic than the original molecule, such as α-zearalenol [98]. Within the previously mentioned additives, adsorbent materials and organic postbiotic components represent the main physical detoxification methods used to separate and neutralize ZEN according to its physical characteristics, including adsorption and extraction [71,101]. However, the highest efficacy in creating ZEN-binding complexes was obtained by YCW since β-D-glucans contained in YCW play a strong role in binding this mycotoxin [67]. Also, the addition of 80 mg/animal of the curcumin extract was reported to improve the antioxidant response and performance parameters in ruminants. However, information on the milk thistle extract is scant in ruminants. Regarding the scientific evidence based on ZEN adsorption by YCW, it has been widely recognized that this matrix can effectively bind ZEN [71,98,102]; in fact, the inclusion of YCW and hydrolyzed yeast in the tested anti-mycotoxin agent allowed us to achieve a weak binding, ranging from 59.2% to 96.4% and a strong binding ranging from 24.9% to 82.5% at different time points.

Regarding DON, this mycotoxin represents one of the mycotoxins with the highest incidence in feed rations for dairy cows [20]. The primary effects include feed intake reduction, ruminal alteration, and energy-corrected milk yield imbalance [103]. It is known that the ruminal detoxification mechanism can reduce the magnitude of mycotoxin’s effects on health. In fact, when this mycotoxin is ingested, around 20–40% is metabolized into a non-toxic de-epoxide metabolite (DOM-1), thus naturally decreasing the DON entering the bloodstream [104,105]. However, it is well known that when the ruminal pH is low, this condition is hampered, and there is an accumulation of DON since no transformation occurs [106]. In this case, the clay showed a significantly low affinity for DON [62]. Thus, different strategies have been developed in order to reduce their concentration. For example, biological detoxification methods based on microbial strains or enzymes have the advantages of high specificity and efficiency [107]. Within this latter category, it has been identified that the cell wall of certain bacteria can absorb this mycotoxin: some studies have shown that the *Saccharomyces cerevisiae* strain reduced the soluble DON content by 39% when added to a PBS buffer contaminated with DON [44]. Also, Konk et al. demonstrated that a yeast cell wall-based product was able to absorb 22.9% of DON [108]. DON is known to increase oxidative stress. At the molecular level, it binds to ribosomes and induces ribotoxic stress, leading to the activation of mitogen-activated protein kinases (MAPKs), cell cycle arrest, and apoptosis [109]. Thus, another approach to minimize these effects is the inclusion of antioxidant agents for the detoxification of DON-contaminated feeds. A previous study investigating the efficacy of this anti-mycotoxin agent in piglets [60] and sows [61], both subjected to multi-mycotoxin contamination, demonstrated significant improvements in the redox status and overall animal performance, which was attributed to the phytogenic extracts included in the final formulation. Our findings are in accordance with Papatsiros et al., where the sequestered percentage through weak binding ranged from 49.1% to 84.0%, demonstrating a wide variability in the binding efficacy [60,61]. Meanwhile, the percentage of DON released by the pellet through strong binding ranged from 1.3% to 41.6%.

Concerning FBs, the primary effect described in dairy cows is a reduction in milk yield and an increase in SCC [110]. However, it has been widely demonstrated that this group of mycotoxins interferes with sphingolipid metabolism, leading to an increase in intracellular-free Sa [111]. As a result, the variation in the Sa/So ratio, marked by elevated Sa in blood and urine samples, can be used as a biomarker for FB’s exposure and effect [112]. The ruminal degradation and intestinal adsorption of FBs are minimal, leading to the predominant excretion of mycotoxin in the feces [113]. Our results show that for FBs, the sequestered percentage through weak binding ranges from 43.4% to 63.6%. The released percentage by the pellet through strong binding varies from 31.0% to 62.8% at different time intervals. However, since FBs have very low oral availability, there has never been a serious problem in ruminants [110]. On the other hand, some authors have shown that the contamination with 22 mg/kg of FBs induced an increase in enzyme activity, thus indicating hepatoxicity and alterations in ruminal function [102,111]. These results highlight the necessity of using protective agents with high antioxidants and hepatoprotective capacity that allow the physiological conditions of the target organs involved to be safeguarded.

Another group of mycotoxins that can be reduced by the same mode of action are T-2 and HT-2 -toxins. In ruminants, these are known to induce immunosuppression, diarrhea, leucopenia, lymphopenia, and the lymphoid depletion of mesenteric lymph nodes and the spleen [114]. However, there is little information on the effectiveness of detoxification solutions for these mycotoxins in the literature. Our findings showed that anti-mycotoxin was highly efficient in binding these two toxins (i.e., the results were always under the LOD for all samples). It should be considered that during in vitro incubation, mycotoxins that were previously adsorbed may be released as particles from the feed degrade. Consequently, the measured concentration of mycotoxins might rise over time, as only the unbound fraction is detected. Taken together, our findings highlight the importance of high initial binding (weak binding) to capture mycotoxins effectively and low desorption (strong binding) to ensure that mycotoxins do not become bioavailable again.

As far as the presence of the yeast cell wall characterizing the tested anti-mycotoxin agent is concerned, some previous studies indicate a positive correlation between the quantity of cell wall material, wall thickness, and cell diameter, with an increased cell wall content enhancing mycotoxin adsorption [69,115,116]. Additionally, these polysaccharides interact directly with immune cells and bind to pathogens, preventing their adhesion to the gastrointestinal tract [117]. In dairy ruminants, there is little evidence of the use of organic components to mitigate the harmful effects of mycotoxins [118,119]. Firmin et al. [118] demonstrated the ability of a modified yeast cell wall extract to reduce AFB1 absorption in dairy ewes. In this study, the yeast cell wall reduced the absorption of AFB1 and increased the elimination of AFB1 and AFM1 in ewe feces up to 100%. Regarding hydrolyzed yeast, this component results from yeast extraction via hydrolysis, comprising both yeast extract and yeast cell wall components, and it has been shown to positively recover intestinal integrity and immune responses after contamination with FBs, AFB1, ZEA, and DON [119,120].

### 2.3. Multivariate Statistics Highlight the Effects of the Anti-Mycotoxin Agent

**Figure 3:**
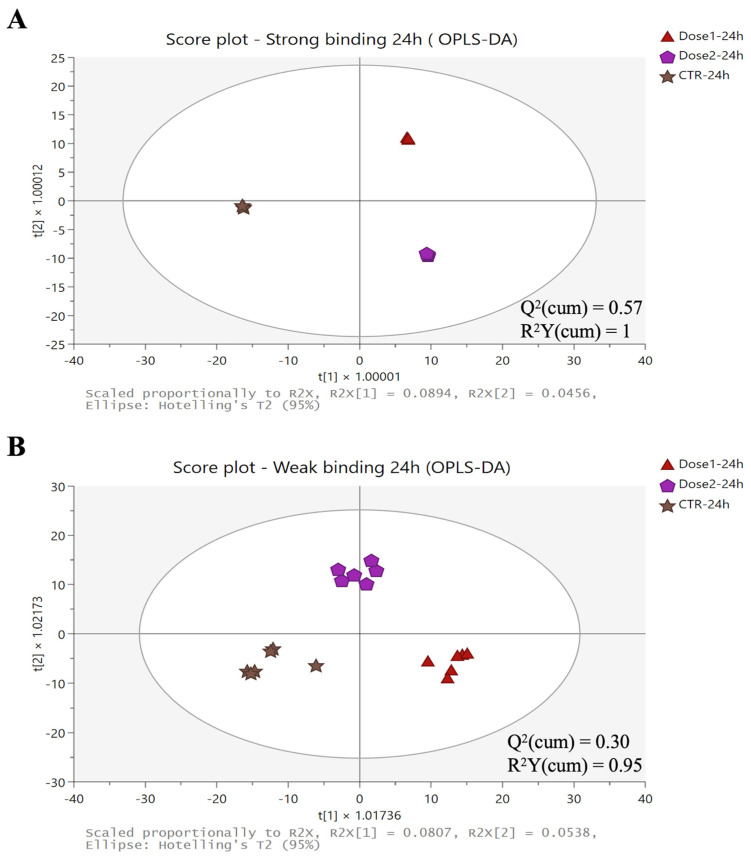
OPLS-DA score plots considering the untargeted chemical profile of rumen samples after 24 h of incubation for both strong (**A**) and weak (**B**) binding experiments.

The untargeted screening carried out on full MS raw data allowed the annotation of more than 1500 mass features (Table S1). This list of mass features was used to carry out both unsupervised and supervised multivariate statistical analyses to better highlight the effect of the anti-mycotoxin agent used. The heat maps (Table S1) resulting from the unsupervised HCA allowed us to observe a clear modification of the metabolomic profile of rumen samples incubated with mycotoxins (CTR) and with different doses of anti-mycotoxin agents (i.e., Dose 1 and Dose 2). As a general consideration, the heat maps showed a clear effect of Dose 1 and Dose 2 after 24 h of incubation, which was significantly different from the CTR sample. These differences were better outlined in rumen samples resulting from strong binding experiments when compared to those from weak binding experiments (Table S1). Also, the weak binding heat map was characterized by two main clusters; the first one included the addition of rumen samples with Dose 1 and Dose 2 at the earlier incubation points (i.e., 1 and 4 h) and the second one included the remaining samples. Considering the output obtained from HCA together with those reported in Table 1, we carried out additional multivariate statistical analyses based on a supervised OPLS-DA to evaluate significant differences between CTR samples and anti-mycotoxin agent-added samples after 24 h of incubation. The corresponding OPLS-DA score plots are reported in Figure 3.

Interestingly, both the OPLS-DA score plots clearly outlined the impact of the anti-mycotoxin agent on the untargeted profile of samples after being incubated for 24 h. A higher prediction ability of the measured mass features was found for the strong binding when compared with the weak binding, recording Q^2^(cum) values of 0.57 and 0.30, respectively. Also, a higher variability between the different sample aliquots was measured for the weak binding when compared to the strong binding group of samples. Overall, both Dose 1 and Dose 2 determined a different chemical profile in the rumen, and this effect was marked for the strong binding results (Figure 3A), while for weak binding, we found a marked between-variability mainly for Dose 1 rumen samples (Figure 3B).

The VIP selection method was then used to list the most discriminant compounds for both OPLS-DA models. Overall, the OPLS-DA model built on strong binding was characterized by 44 highly discriminant compounds, while that built on weak binding revealed a lower number of compounds (16). These metabolites are reported in Table 2, together with their VIP score and Log_2_FC values for the pairwise comparisons of Dose 1 vs. CTR and Dose 2 vs. CTR.

Looking at the VIP metabolites, we found seven shared compounds between the weak and strong binding (in terms of prediction ability), namely deepoxy-deoxynivalenol, fumonisin B1, mycophenolic acid, quercetin 3,7-dimethyl ether, ellagic acid, diosmin, and formononetin. Interestingly, among the exclusive marker compounds of weak binding, we found (among the others) silibinin; this latter compound (also known as silybin) is the most active component of silymarin, which is a flavonolignan complex extracted from the seeds of the milk thistle plant (*Silybum marianum*). Silibinin is primarily known for its antioxidant, anti-inflammatory, and hepatoprotective properties. Additionally, we found several phenolic compounds belonging to the class of flavonoids (such as flavones and flavonols), together with some carotenoids, tocopherols, and additional mycotoxins and emerging fungal metabolites (Table 2). As a general consideration, we found a higher number of VIP discriminant compounds for the strong binding group, including several mycotoxins, such as AFB1, deepoxy-nivalenol, emodin, FBs (A1, B1, B2, B3, B4), HT-2, mycophenolic acid, nivalenol, roquefortine A, altertoxin I, fusidic acid, and zearalenone. Looking at the Log_2_FC values for strong binding, AFB1 was up-accumulated following 24 h of incubation in the Dose 1 and Dose 2 groups when compared with CTR samples, recording values of 0.740 and 0.976, respectively. Also, the group of FBs showed an overall down-accumulation when comparing Dose 1 and Dose 2 with the CTR samples (Table 2). As far as HT-2 and zearalenone are concerned, they both showed a down-accumulation of values when comparing both anti-mycotoxin agent groups with CTR samples. Therefore, taken together, our untargeted data confirmed what was previously postulated by looking at the % of sequestered and released mycotoxins, suggesting the pivotal role exerted by the phytogenic extracts in modulating the effect of the anti-mycotoxin product.

## 3. Conclusions

This study demonstrates the potential efficacy of a novel anti-mycotoxin agent in mitigating mycotoxin contamination in ruminants using an in vitro ruminal model. The product, which combines adsorbing material, phytogenic extracts, and post-biotic compounds, exhibited a multi-faceted mode of action that effectively bound several mycotoxins, including AFB1, T-2, and HT-2 toxins, with a high binding efficiency. The agent’s ability to sequester ZEN, DON, and FBs was also noteworthy, with variations observed in weak and strong binding efficiencies across different incubation times, with marker differences emerging after 24 h of incubation. These findings highlight the agent’s capability to provide substantial protection against mycotoxins by ensuring high initial sequestration and relatively low desorption rates, thus supporting its use as a valuable tool in ruminant feed management. The fluctuating strong binding values observed were likely related to the complex interplay of factors, such as the mycotoxin structure, pH variation, binding interaction types, the competition among toxins, matrix effects, and binding kinetics. These findings emphasize the importance of considering both the binding strength and the stability of interactions over time, suggesting that this product could play a crucial role in safeguarding ruminant health and production, particularly under conditions where ruminal detoxification may be compromised.

## 4. Materials and Methods

The sequestering agent was evaluated for its capacity to adsorb and/or bio-convert mycotoxins by adopting a rumen simulation method. The groups of mycotoxins tested in the current trial were aflatoxins (AFs), fumonisins B1 and B2 (FBs), trichothecene deoxynivalenol (DON), T-2 and HT-2, and zearalenone (ZEN). To assess the agent’s efficacy, two doses were tested in vitro based on a constant rumen volume of 50 L under laboratory conditions:

- Dose 1 simulated 30 mg per cow per day of the anti-mycotoxin agent;
- Dose 2 simulated 90 mg per cow per day of the anti-mycotoxin agent.

The concentration of each mycotoxin, expressed as parts per million (ppm) on a dry matter dietary basis, is reported in Table 3.

### 4.1. Contaminated Starter for the In Vitro Trials

The AFB1 starter was derived from a naturally contaminated corn meal, with a final concentration of 0.0239 mg/kg DM (ppm). The FBs, DON, and ZEN starters (from mycotoxin-contaminated culture media) had final concentrations of 17.7 mg/kg DM, 3.3 mg/kg DM, and 4.42 mg/kg DM, respectively. The concentrations of T-2 and HT-2 toxins were below the limit of detection (1 part per billion, ppb) and are, therefore, not reported.

### 4.2. Preparation of Mycotoxins Solutions

Mycotoxins were extracted individually from the respective solid matrices. The extracting solution (2 L) consisted of 1475 mL of acetonitrile (ACN; HPLC grade), 500 mL of H_2_O (HPLC grade), and 25 mL of glacial acetic acid. The extractions were conducted using 5 g of the matrices and 40 mL of the extraction solution at room temperature with gentle oscillation for 20 min. The mixture was then centrifuged at 5500 rpm for 5 min to precipitate the pellet. Samples were subsequently frozen at −20 °C overnight. If the pellet was resuspended, a second centrifugation step was performed. The supernatant was then recovered to prepare the final contaminated solution. The mycotoxin concentrations obtained for each extract were 8.38 μg/L (ppb) for AFB1, 4775.9 ppb for FB, 825 ppb for DON, and 1105 ppb for ZEN.

### 4.3. In Vitro Experimental Design

Previously, a uniform mixture of a corn–barley-flaked diet was collected and dried at 65 °C in an oven until it reached a constant weight. Then, it was ground to a particle size of 1 mm using a laboratory mill with adjustable speed (Pullverisette 19) and used for the substrate in the in vitro gas production assay.

The in vitro gas production tests were set up using 100 mL glass bottles that contained a mixture of ~220 mg of corn–barley meal, 10 mL of rumen fluid, and 20 mL of a buffer solution that simulates the electrolyte composition of rumen fluid. The treatment used in the in vitro experiment was conducted as follows:

- Control: the substrate of corn–barley meal with the inoculum of mycotoxins;
- Dose 1: the substrate of corn–barley meal with the inoculum of mycotoxins and a low dose of the anti-mycotoxin agent (30 mg/cow/d);
- Dose 2: the substrate of corn–barley meal with the inoculum of mycotoxins and a high dose of the anti-mycotoxin agent (90 mg/cow/d).

For each treatment, there were 3 analytical replicates at each incubation time (1, 4, and 24 h post-incubation) and 2 experimental runs useful to obtain experimental replicates for a total of 3 treatments × 3 analytical replicates × 3 incubation times × 2 incubation runs = 54 samples. The dynamics of gas production were verified by adding 2 bottles with only buffered rumen fluid (blanks) and 2 bottles with a starch sample for an internal standard (Gelose 80 maize starch; Penford Food Ingredients Co., Centennial, CO, USA) in each incubation. The cumulative gas production from the blanks and internal standards was averaged, and it was considered satisfactory if it fell within ±1 standard deviation.

### 4.4. Rumen Fluid Collection

Rumen fluids were obtained through manual collection from two Holstein dry dairy cows equipped with ruminal fistulas (average body weight 625 ± 10 kg), farmed at the CERZOO experimental station located in San Bonico, Piacenza, Italy. The cows were provided with maintenance diets as part of their daily regimen, and samples of rumen content were meticulously gathered after the morning feeding session in accordance with the guidelines outlined by the National Research Council in 2001.

The time between rumen fluid collection and the inoculum in vitro was kept to a minimum of 20 min to prevent any changes in microbial activity and fermentation. This was guaranteed with the use of thermos during transportation to the laboratory, and after rapid filtering through four layers of cheesecloth, the rumen fluid was added with a buffer–mineral solution. The procedure to prepare the buffer–mineral solution was described by Menke and Steingass [121]. On the same day of the fermentation, it was prepared and maintained at 39 °C in a water bath under anaerobic conditions with continuous CO_2_ flushing and an adjusted pH of around 6.5 to 6.6. The collected rumen fluids were diluted in a 1:2 volume ratio of rumen fluid to the buffer–mineral solution. Continuous agitation and CO_2_ flushing were conducted to ensure anaerobic conditions at 39 °C were maintained.

### 4.5. Inoculum Procedure

An adjustment procedure used to process the buffered rumen fluid was described by Pirondini et al. and Serment et al. (2016), where a volume of 30 mL of diluted rumen fluid was added to each glass bottle [122,123]. Then, calculated aliquots of each solution contaminated with mycotoxins were added with a graduated pipette in order to guarantee the previously reported dose. Then, the headspace of the bottle (with a volume of 70 mL) was flushed with CO_2_ and then hermetically closed with rubber caps for degassing. All the bottles were incubated in a thermal water bath, and the pressure in the headspace was measured at intervals of 1, 4, and 24 h using a digital test gauge XP2i by the Crystal Engineering Corp. After the measurement, the gas was then released by a sterile needle, and the pressure was brought back to normal levels. To maintain regular microbial activity, the headspace pressure never went beyond 48 kPa in accordance with Theodorou et al.’s (1994) recommendation [124]. The absolute pressure observed was added up to determine the cumulative gas production and was then converted to moles of gas using the ideal gas law:

n = p × V/(R × T)

where n is the moles of gas, p is pressure (psi), V is the headspace volume (L), R is the gas constant (8.314 L·kPa·K^–1^·mol^–1^), and T is the temperature (K).

### 4.6. Extraction Step and Multiscreening of Mycotoxins

The replicate glass bottles at the respective hours of incubation were removed from the thermal bath and stored at −20 °C before the determination of the mycotoxins. Briefly, samples were thawed at room temperature and centrifuged in 50 mL falcon tubes at 5500 rpm to promote the precipitation of suspended material. An aliquot of 200 μL of the supernatant was collected and added with a volume of 800 μL of the extraction solution, consisting of ACN/H_2_O HPLC grade/glacial acetic acid (73.75/25/1.25, *v*/*v*/*v*). The remaining pellet was weighed before the next extraction step. This latter step was performed for the evaluation of strong binding, i.e., stability of the complex anti-mycotoxin agent-mycotoxins. To perform this, the solutions were shaken by vortexing for 1 minute, and then an ultrasound-assisted extraction was performed on all samples at maximum power (120 Watt) for 5 min at room temperature. Samples were centrifuged at the maximum speed (5500 rpm), setting a temperature of 4 °C for 15 min. Finally, the extracts were filtered using RC-syringe filters of 0.22 μm and stored in an amber vial for the UHPLC-HRMS analysis.

The analyses were performed using a UHPLC instrument (Dionex Ultimate 3000; Thermo Fisher Scientific, Waltham, MA, USA) coupled with a Q-Exactive Focus Orbitrap mass spectrometer (Thermo Fisher Scientific). The UHPLC system consisted of a degassing system, a quaternary UHPLC pump, an autosampler device, and a thermostatically controlled Thermo Scientific™ Hypersil GOLD™ aQ (100 × 2.1 mm, 1.9 μm) held at 35 °C. The mobile phase consisted of (A) water with 0.1% formic acid, 2% methanol, containing 5 mM ammonium formate, and (B) methanol with 0.1% formic acid, 2% water, and containing 5 mM ammonium formate. A gradient elution program was applied as follows: an initial 0% B was held for 0.5 min, increased to 100% B over 7.5 min, and held for 0.5 min. Then, the gradient was decreased to 0% B over 6 min to re-equilibrate the instrument for a total run time of 15 min. The flow rate was 0.3 mL/min, while the injection volume was 3 μL. Detection was performed using a Q-Exactive Focus Orbitrap mass spectrometer. The mass spectrometer was operated in the positive and negative ion mode by setting 2 scan events: full ion MS and parallel reaction monitoring (PRM) for targeted fragmentation. Full scan data were acquired at a resolving power of 70,000 for full width at half maximum at 200 *m*/*z*. The mass range in the full scan experiments was set at 250–850 *m*/*z*. The conditions in the positive ionization mode (ESI+) were as follows: spray voltage 3500 V; capillary temperature 320 °C; S-lens RF level 50; sheath gas pressure (N_2_ > 95%) 40; auxiliary gas (N_2_ > 95%) 20; and auxiliary gas heater temperature 320 °C. The conditions in the negative ionization mode (ESI−) were as follows: spray voltage 2800 V; capillary temperature 320 °C; S-lens RF level 50; sheath gas pressure (N_2_ > 95%) 35; auxiliary gas (N_2_ > 95%) 15; and auxiliary gas heater temperature 320 °C. The parameters for the scan event of PRM were as follows: a mass resolving power of 17,500 for full width at half maximum (200 *m*/*z*), an AGC target at 2 × 10^5^, a maximum IT at 100 ms, and an isolation window at 2.0 *m*/*z* for accurate mass measurement fragments. Calibration curves for the mycotoxins targeted were prepared in the range of 1–1000 μg/L (ppb), with a coefficient of determination (R^2^) for MSMS > 98%. The following mycotoxins were targeted: aflatoxin B1 (fragments range: 285–314 Da), fumonisin B1 (fragments range: 352–353 Da), fumonisin B2 and B3 (fragments range: 336–337 Da), deoxynivalenol (fragments range: 177–250 Da), H-T2 (fragments range: 216–264 Da), T2 (fragments range: 185–306 Da), and zearalenone (fragment range: 272–319 Da). Standards of different mycotoxins (purity > 98%) were purchased from Sigma-Aldrich (St. Louis, MA, USA) and VWR (Radnor, PA, USA).

The untargeted screening was carried out on the same full-scan MS raw data, using the software MS-DIAL (version 4.90) for data elaboration [125]. The mass range 250–850 *m*/*z* was searched for features with a minimum peak height of 10,000 cps. The MS and MS/MS tolerance for peak centroiding were set to 0.05 and 0.1 Da, respectively. The accurate mass tolerance for identification was 0.05 Da for MS and 0.1 Da for MS/MS. The identification step was based on mass accuracy, the isotopic pattern, and spectral matching. In MS-DIAL, these criteria were used to calculate the total identification score. The total identification score cut-off was >50%, considering the most common ESI+ adducts. Gap filling using the peak finder algorithm was performed to fill in the missing peaks, considering 5 ppm tolerance for *m*/*z* values. The ESI-positive MSMS library of MS-DIAL was coupled with a custom database containing a list of mycotoxins and main metabolites for tentative annotation according to the accurate mass and isotopic profile of each compound.

### 4.7. Statistical Analysis

The fermentation runs were completed in sequenced periods, and data from two fermentation bottles within each run were averaged and used as statistical units (replicates among the runs). Statistical analysis was then performed with the general linear model (GLM) procedure using R software (version 4.4.1). Data were statistically analyzed as part of a completely randomized design with a factorial arrangement of treatments using the following model:

Y = μ + Treatment + Time + Treatment × Time + e

where ‘Y’ is the experimental data (response variable), ‘μ’ is the overall mean, ‘Treatment’ represents the fixed effect of the treatments (CTR, Dose 1 and Dose 2), ‘Time’ represents the fixed effect of the time points (1,4, and 24 h), ‘Treatment × Time” is the interaction term between treatment (CTR, Dose 1 and Dose 2) and time (1, 4, and 24 h) to assess if the effect of the treatment differs across time points, and ‘e’ is the residual error term. Also, examining the linear and quadratic effects of the treatment as factors (considering a 3-level factor, i.e., CTR, Dose 1, and Dose 2), orthogonal contrasts were performed using the same software. In this way, it is possible to assess whether there is a linear or quadratic effect across the increasing doses of the anti-mycotoxin agent. Finally, estimated marginal means (emmean), defined as the marginal (weighed) means of model predictions over the grid comprising all factor combinations, was used to assess significant differences (*p* < 0.05) between the different treatments.

The high-resolution mass spectrometry (HRMS) data were then elaborated using the software MetaboAnalyst 5.0 and SIMCA (Umetrics, Sweden) [125]. Briefly, after data normalization, both unsupervised and supervised multivariate statistics were calculated. The unsupervised approach was based on hierarchical cluster analysis (HCA; Table S1), while the orthogonal projections to latent structure discriminant analysis (OPLS-DA) were used as the supervised tool. Additionally, the OPLS-DA model validation parameters (goodness-of-fit R^2^Y together with goodness-of-prediction Q^2^Y) were inspected, considering a Q^2^Y prediction ability of >0.5 as the acceptability threshold. Thereafter, the OPLS-DA model produced was inspected for outliers, and permutation testing (N > 100) was performed to exclude model over-fitting. The importance of each mycotoxin was detected as resulting from weak and strong binding experiments for discrimination purposes and was then calculated according to the variable selection method VIP (i.e., variables importance in projection), considering values higher than 1 as the minimum significant threshold, and also calculating the log_2_ fold-change value for the pairwise comparisons of Dose 1 and Dose 2 vs. the CTR.

## Figures and Tables

**Table 1:** Initial sequestration (weak binding) and desorption (strong binding) of the anti-mycotoxin agent for the studied mycotoxins.

		Treatments									
		CTR			Dose 1			Dose 2			
**Mycotoxin**		1 h	4 h	24 h	1 h	4 h	24 h	1 h	4 h	24 h	RMSE
ZEN	% Sequestered *	59.2	63.1	64.2	92.8	94.7	96.3	94.9	95.2	96.3	0.016
	mean		62.1 ^a^			94.6 ^b^			95.3 ^b^		
	% Released **	67.4	71.8	50.4	82.4	77.3	27.7	76.4	60.6	24.8	0.250
	mean		63.2 ^a^			62.4 ^a^			54.0 ^a^		
DON	% Sequestered *	49.0	49.4	59.8	73.7	72.5	83.9	73.3	78.7	85.4	0.072
	mean		52.8 ^a^			76.9 ^b^			79.2 ^b^		
	% Released *	7.1	9.9	1.3	7.1	7.8	2.6	6.2	11.9	3.8	0.048
	mean		5.9 ^a^			6.0 ^a^			7.4 ^a^		
FBs	% Sequestered *	44.9	44.3	43.4	60.1	62.7	63.6	63.2	59.7	53.1	0.058
	mean		44.2 ^a^			58.7 ^b^			62.1 ^b^		
	% Released **	49.1	62.8	62.2	32.7	32.2	31.9	31.0	41.5	59.1	0.127
	mean		58.1 ^b^			32.3 ^a^			43.9 ^a^		

* = % Sequestered (weak binding); ** = % Released (strong binding). Abbreviations: RMSE, root mean square error; mean, estimated marginal mean (at a weighted time point). Estimated marginal means with different superscript letters (^a,b^) differ at *p* < 0.05.

**Table 2:** Most discriminant VIP metabolites together with their variation for both weak and strong binding experiments. ns = not significant.

VIP Weak Binding	VIP Score	Log_2_FC Dose 1 vs. CTR	Log_2_FC Dose 2 vs. CTR
Alternariol	1.183	−0.465	ns
Apicidin	1.318	ns	−0.324
Beauvericin	1.223	−4.51	−1.74
Deepoxy-deoxynivalenol	1.073	0.214	0.160
Fumonisin B1	1.166	−0.347	0.343
Mycophenolic acid	1.142	0.484	0.436
Citrinin	1.039	0.361	−0.864
Lycopene	1.094	0.594	0.169
Silibinin	1.112	ns	0.417
Formononetin	1.125	0.202	0.276
Diosmin	1.109	1.20	−1.56
Quercetin 3,7-dimethyl ether	1.157	ns	0.448
2-(2,3-dihydrobenzo[b][1,4]dioxin-6-yl)-6-nitro-4H-chromen-4-one	1.233	0.179	ns
Ellagic acid	1.357	0.191	ns
Genistin	1.361	−0.757	ns
VIP strong binding	VIP score	Log_2_FC Dose 1 Vs. CTR	Log_2_FC Dose 2 Vs. CTR
AFB1	1.339	0.740	0.976
Deepoxy-deoxynivalenol	1.395	0.612	0.653
Emodin	1.088	0.381	0.409
Enniatin B1	1.010	ns	0.446
Fumonisin A1	1.003	−0.577	−0.306
Fumonisin B1	1.146	−0.675	0.149
Fumonisin B2	1.062	−0.668	−0.240
Fumonisin B3	1.154	−0.354	−0.192
Fumonisin B4	1.078	−0.170	ns
HT-2	1.211	−0.608	−0.365
Mycophenolic acid	1.192	0.284	0.456
Nivalenol	1.228	ns	0.297
Zearalenone	1.133	−0.872	−0.953
Apigenin	1.015	ns	−0.248
beta, beta-carotene	1.023	−0.342	ns
6-methoxy-7-(3-methylbut-2-enoxy)chromen-2-one	1.036	−0.391	−0.462
Adenosine-3-monophosphate	1.052	−0.954	−0.206
delta-tocopherol	1.077	0.228	0.687
Quercetin 3,7-dimethyl ether	1.080	0.342	0.405
Roquefortine A	1.108	ns	−0.205
Formononetin	1.126	0.179	ns
Genistein	1.144	−0.297	−0.285
Schisandrin	1.153	1.574	1.124
Altertoxin I	1.155	1.726	1.499
(-)-Gallocatechin-3-O-gallate	1.167	ns	0.332
Sphinganine 1-phosphate	1.173	ns	−0.146
PC(16:0/18:1(9Z))	1.175	0.316	ns
Kaempferol	1.184	−1.143	−1.378
LPC 18:2	1.209	1.276	1.628
Poncirin	1.223	0.335	−0.314
Phosphatidylethanolamine (20:3/16:1)	1.246	ns	−0.279
Naringenin	1.259	−0.496	0.394
4-methylumbelliferyl acetate	1.289	0.210	0.341
Neoeriocitrin	1.309	2.354	2.515
Ellagic acid	1.355	ns	0.293
3,7-Dihydroxyflavone	1.359	0.226	ns
Sphingosine-1-Phosphate (C17 base)	1.402	0.530	0.486
Fusidic acid	1.427	−0.351	−0.384
Quercetin-3-O-beta-galactoside	1.482	−0.764	−1.598
4′-hydroxy-2′,4,6′-trimethoxychalcone	1.494	0.350	0.319
alpha-Tocopherol	1.524	0.191	−0.291
Naringenin-7-O-glucoside	1.527	−0.144	−0.418
Chrysin	1.533	1.148	1.023
Diosmin	1.687	1.119	1.929

**Table 3:** Mycotoxin concentrations to assess the efficacy of the sequestering agent.

Mycotoxins	mg/kg DM
Aflatoxins	0.002
Fumonisins	10
Deoxynivalenol	2
Zearalenone	1
T2 and H-T2	1

## Data Availability

The original contributions presented in the study are included in the article. Further inquiries can be directed to the corresponding author.

## Supplementary Materials

The following supporting information can be downloaded at https://www.mdpi.com/article/10.3390/toxins16110490/s1. Table S1: Excel file containing the mass features annotated by UHPLC-HRMS in rumen samples, together with the statistical analyses carried out on both strong and weak binding data.
